# Added value of advanced over conventional magnetic resonance imaging in grading gliomas and other primary brain tumors

**DOI:** 10.1186/s40644-014-0035-8

**Published:** 2014-12-12

**Authors:** Juan A Guzmán-De-Villoria, José M Mateos-Pérez, Pilar Fernández-García, Enrique Castro, Manuel Desco

**Affiliations:** Servicio de Radiodiagnóstico. Hospital General Universitario Gregorio Marañón, Madrid, Spain; Centro de Investigación Biomédica en Red de Salud Mental (CIBERSAM), Madrid, Spain; Instituto de Investigación Sanitaria Gregorio Marañón, Madrid, Spain; Departamento de Bioingeniería e Ingeniería Aeroespacial, Universidad Carlos III, Madrid, Spain

**Keywords:** Brain neoplasms, Magnetic resonance imaging, Magnetic resonance spectroscopy, Diffusion-weighted MRI, Perfusion-weighted MRI

## Abstract

**Background:**

Although conventional MR imaging (MRI) is the most widely used non-invasive technique for brain tumor grading, its accuracy has been reported to be relatively low. Advanced MR techniques, such as perfusion-weighted imaging (PWI), diffusion-weighted imaging (DWI), and magnetic resonance spectroscopy (MRS), could predict neoplastic histology, but their added value over conventional MRI is still open to debate.

**Methods:**

We prospectively analyzed 129 patients diagnosed with primary brain tumors (118 gliomas) classified as low-grade in 30 cases and high-grade in 99 cases.

**Results:**

Significant differences were obtained in high-grade tumors for conventional MRI variables (necrosis, enhancement, edema, hemorrhage, and neovascularization); high relative cerebral blood volume values (rCBV), low relative apparent diffusion coefficients (rADC), high ratio of N-acetyl-aspartate/creatine at short echo time (TE) and high choline/creatine at long TE. Among conventional MRI variables, the presence of enhancement and necrosis were demonstrated to be the best predictors of high grade in primary brain tumors (sensitivity 95.9%; specificity 70%). The best results in primary brain tumors were obtained for enhancement, necrosis, and rADC (sensitivity 98.9%; specificity 75.9%). Necrosis and enhancement were the only predictors of high grade in gliomas (sensitivity 97.6%; specificity 76%) when all the magnetic resonance variables were combined.

**Conclusions:**

MRI is highly accurate in the assessment of tumor grade. The combination of conventional MRI features with advanced MR variables showed only improved tumor grading by adding rADC to conventional MRI variables in primary brain tumors.

## Background

Primary brain tumors constitute a heterogeneous group that can be classified according to their histological type and grade of malignancy. The World Health Organization (WHO) classifies primary brain tumors into four different grades of malignancy [1]. Histological tumor grading has several drawbacks, one of which is the need for stereotactic biopsy, an invasive procedure with a certain risk of morbidity and mortality. In addition, this approach is subject to sampling error, and its results depend upon the neuropathologist’s experience [2]. These limitations lend support to research into non-invasive imaging techniques.

Although conventional Magnetic Resonance Imaging (MRI) is an established technique for the characterization of brain tumors, it is not completely reliable [3]. Perfusion Weighted Imaging (PWI), diffusion-weighted imaging (DWI), Magnetic Resonance Spectroscopy (MRS) could provide additional information to conventional MRI, as they better reflect histopathology findings [3,4].

The feasibility of PWI, DWI, and MRS for tumor grading has been clearly proved [5–7]. However, their additional value, separately or in different combinations, over conventional MRI has not yet been quantified.

The results obtained with different MR techniques are contradictory, as shown for MRS and PWI with respect to diagnostic accuracy in grading tumors [3,4,8–11]. Furthermore, no significant differences have been found in the assessment of tumor grade using advanced techniques such as PWI [12], MRS [13,14], and DWI [9,15]. Although a small number of studies compared these techniques, to our knowledge only one published study has combined all four in a single center [16].

We hypothesized conventional MRI could accurately evaluate the grade of intraaxial brain tumors, and the added value of other MRI techniques is very small. Our aim was to quantify the improvement in diagnostic accuracy resulting from the combination of conventional MRI with PWI, DWI, and MRS.

## Methods

The study population comprised 129 patients (71 men and 58 women; mean age 52.7 years, range 11 to 84 years) diagnosed with primary brain tumors (the only inclusion criterion) who were consecutively recruited between February 2004 and April 2009 at our institution. The exclusion criteria were as follows: presence of non-neoplastic brain masses; absence of histopathology data; extensive hemorrhage that prevented evaluation by PWI, DWI, and MRS; and previous surgical intervention, chemotherapy, or radiotherapy.

The institutional research and ethics boards of Hospital General Universitario Gregorio Marañón approved the study, and all the patients gave their written informed consent.

### Tumor histology

The histology specimen was obtained by surgical resection in 119 cases and by stereotactic biopsy in 10 cases and analyzed by an expert neuropathologist with more than 30 years of experience blinded to radiological assessment.

Brain tumors were classified as aggressive high-grade tumors (WHO grades III and IV) in 99 cases and low-grade tumors (WHO grades I and II) in the remaining 30 patients (Table 1). We considered grades I and II as low-grade tumors and grades III and IV as high-grade tumors according to the differences in treatment and survival between groups: in general, high-grade tumors present lower survival rates and need complementary therapy after surgery (usually chemotherapy and radiotherapy), whereas in low-grade tumors, survival is higher and the use of complementary therapy remains open to debate.

**Table 1:** **Frequency distribution of histological subtypes of brain tumors**

**Histological subtype**	**WHO grade**	**No.**	**Percentage**
Astrocytoma	I	1	82.9%
	II	16	
	III	20	
	IV	70	
Oligodendroglioma	II	9	8.5%
	III	2	
PNET	IV	7	5.4%
DNET	I	2	1.6%
Hemangioblastoma	I	1	0.8%
Neurocytoma	II	1	0.8%

DNET, dysembryoplastic neuroepithelial tumor; PNET, primitive neuroectodermal tumor.

In order to detect possible misclassifications of histological grading, three months after the end of recruitment medical records were reviewed to determine survival, defined as the time elapsed between MRI diagnosis and death or the last admission to our institution. No case of disagreement was found between survival and tumoral grade that indicates histological misclassifications.

### Conventional MRI

All patients were prospectively examined using a 1.5T MRI scanner (Intera or Achieva, Philips Healthcare, The Netherlands). Sagittal T1-weighted images (647/15 ms [TR/TE] SE) and coronal T2-weighted images (TR 4742/TE, 100 ms; Turbo SE; Fluid-Attenuated Inversion Recovery (FLAIR), 11000/140/2800 ms [TR/TE/TI]) were obtained with a 230-mm, Field Of View (FOV) and matrix size of 512 × 512.

After intravenous administration of a double dose (0.2 mmol/kg) of gadobutrol 1.0 mmol/ml (Gadovist, Bayer Schering Farma, Berlin, Germany), an axial T1-weighted 3D fast-field echo sequence was acquired (TR 16/TE 4.6 ms) with a flip angle of 8°, FOV of 256 × 256 mm, and matrix size of 176 × 288.

### Dynamic contrast-enhanced PWI

PWI was performed using a dynamic contrast-enhanced T2*-weighted gradient echo. EPI- Echo planar images -EPI- (single shot [TR 1678/TE 30 ms] with an EPI factor of 61, flip angle of 40°, matrix size of 128 × 128, and FOV of 230 mm) were acquired during the first pass of the gadobutrol bolus, which was injected intravenously using a 20-gauge needle at a rate of 4.0 ml/sec.

A series of 40 acquisitions were performed at 1.7-second intervals. The first acquisition was acquired before injection to establish baseline (precontrast) intensity.

The results were transferred to a PC workstation for processing (ViewForum Workstation, release 5.1V1L2; Philips Healthcare). The possible effect of tracer recirculation or leakage due to the disruption of the blood–brain barrier was considered in the mathematical model by fitting a gamma-variate function to the observed 1/T2* relaxation rate curve. This gamma-variate function was automatically implemented by the workstation.

### DWI

Diffusion-weighted images were obtained with axial multislice single-shot EPI SE sequences as follows: TR 3745 ms/TE 120 ms; EPI factor, 61; matrix size, 128 × 128; FOV, 230 mm; and diffusion gradient encoding in three orthogonal directions. The images and Apparent Diffusion Coefficient (ADC) maps were calculated using *b* values of 0 and 2500 s/mm^2^. ADC values were quantified using the PC workstation mentioned above.

### MRS

Single-voxel proton MRS was performed in 117 patients. Twelve patients refused to undergo this technique because of the additional examination time involved. The technique used was point-resolved spectroscopy (PRESS) with a TR of 2000 ms and two different TEs (23/144 ms). The measurement of each spectrum was repeated 128 times with a cycling-phase of 16 to improve the signal-to-noise ratio.

The size (mean 8.34 cc, range 5.6 – 18.2 cc) and location of the voxels of interest were established in order to position the largest possible voxel within the solid tumor area, with minimal contamination from the surrounding non-tumor tissue and avoiding areas of necrosis, cysts, or hemorrhage as much as possible. We selected single-voxel MRS owing to its lower time requirements, which enabled all the MR sequences to be performed in a single session.

Spectra were analyzed using custom-designed software [17]. Signal intensity of metabolic peaks, spectral positions, and decay constants were taken into consideration in coupled metabolite peaks. Signals of Choline (Cho), N-acetyl-aspartate (NAA), Creatine (Cr), Lipids (Lip), and Lactate (Lact) were quantified. The same quantification procedure was followed to analyze the water peak, although, in this case, Hankel singular value decomposition was not performed to suppress the water signal.

### Definition of image variables

**Figure 1:**
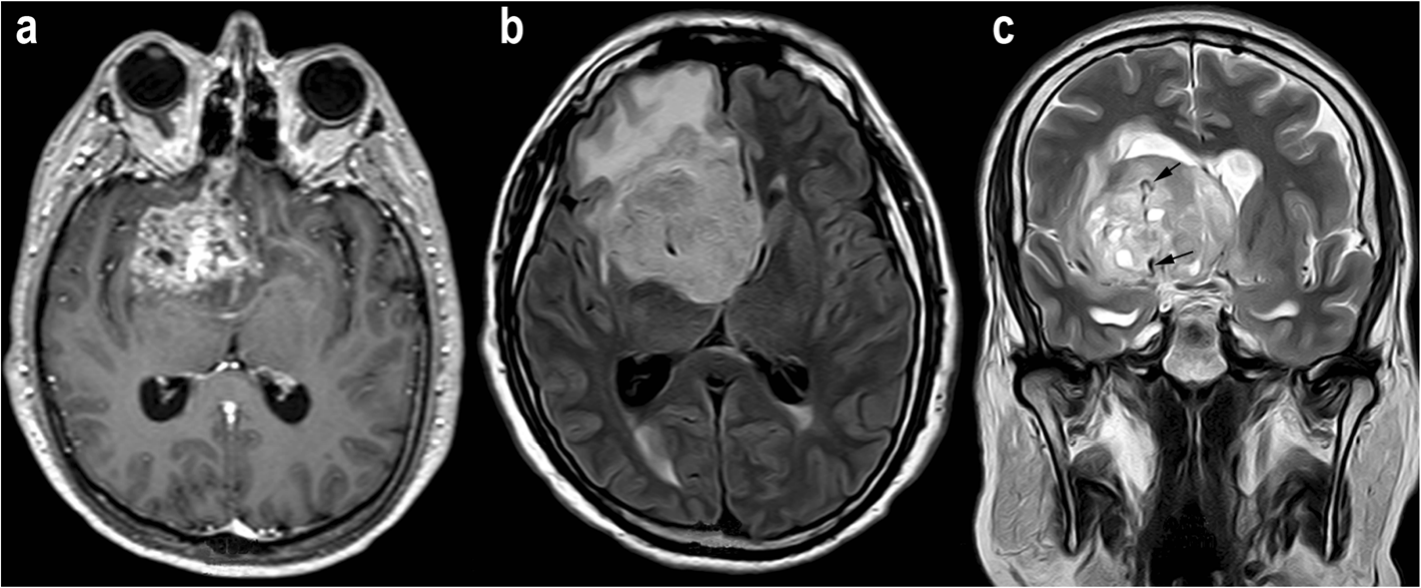
**Example of MR images from a patient with glioblastoma (grade IV). a**, Contrast-enhanced axial T1-weighted image shows an enhancing mass in the right frontal region. **b**, Axial FLAIR image shows peritumoral edema. **c**, Coronal turbo SE T2-weighted image revealing abnormal macroscopic vessels (arrows) within the tumor (neovascularization).

**Figure 2:**
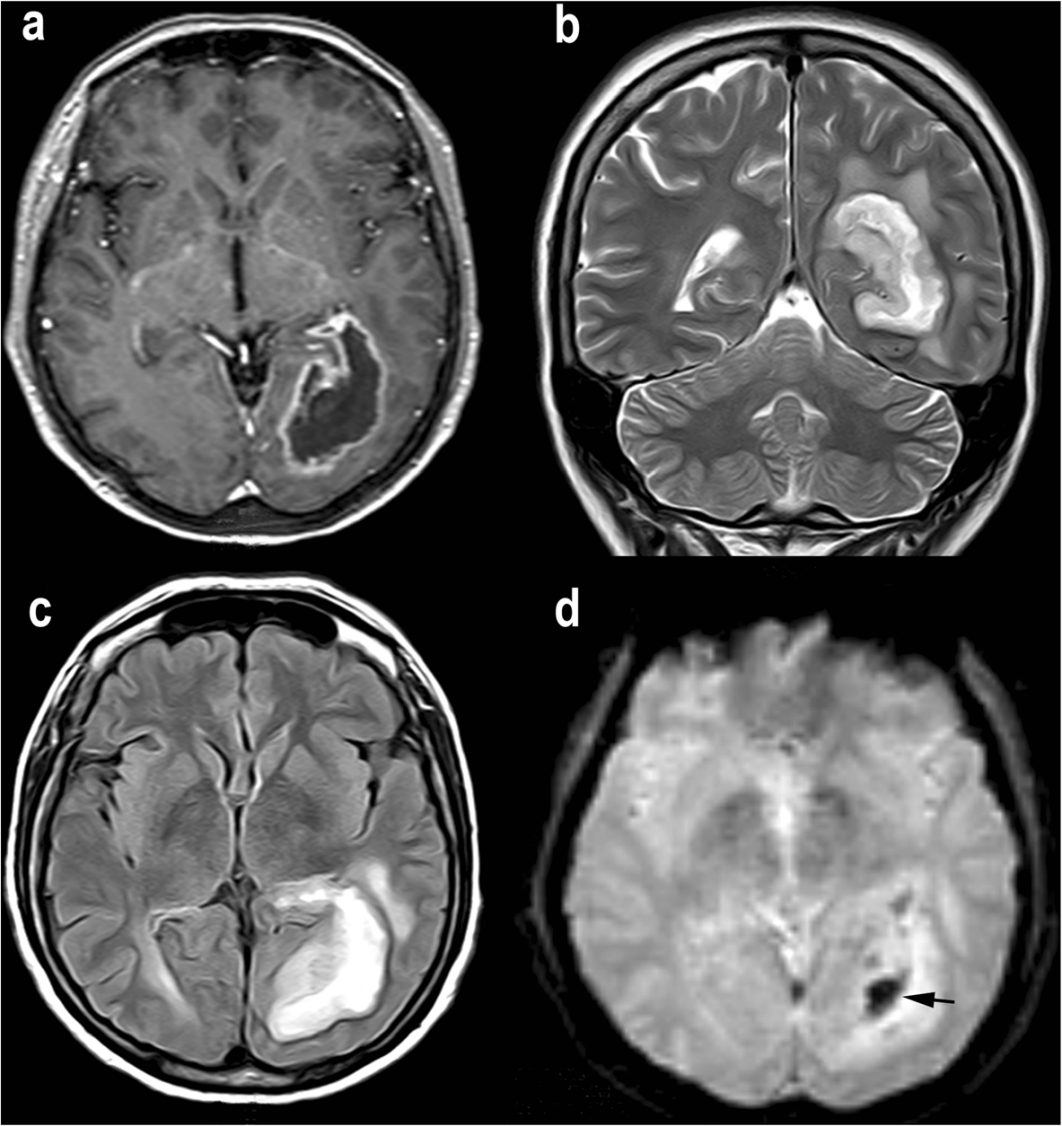
**Example of MR images of glioblastoma (grade IV). a-c**, Signs of necrosis, identified as regions within the neoplasm with hypointensity on contrast-enhanced axial T1-weighted images **(a)** and hyperintensity on T2-weighted images **(b)** and FLAIR images **(c)**. **d**, Signs of hemorrhage seen as an area of hypointensity (arrow) due the paramagnetic blood breakdown products on the axial EPI T2*-weighted images.

The five features evaluated by conventional MRI were as follows: 1) Enhancement, defined as an increased signal in T1-weighted sequences in the tumor after administration of gadolinium; 2) Necrosis (and cystic necrosis), identified as areas within the neoplasm with a signal in T1- and T2-weighted images similar to that of cerebrospinal fluid (on FLAIR images these areas may be hyperintense, owing to excess protein content); 3) Edema, defined as an area of homogenous high signal on FLAIR sequences surrounding the tumor; 4) Neovascularization, defined as the presence of tubular structures within the tumor showing flow-void patterns on T2-weighted images representing abnormal tumor vessels; and 5) Hemorrhage, characterized by an area of magnetic field distortion due to the paramagnetic blood breakdown products on the EPI T2*-weighted images, which were obtained in the first series of PWI before gadolinium reached the cerebral parenchyma (Figures 1 and 2). All five features were assessed dichotomously (presence or absence).

**Figure 3:**
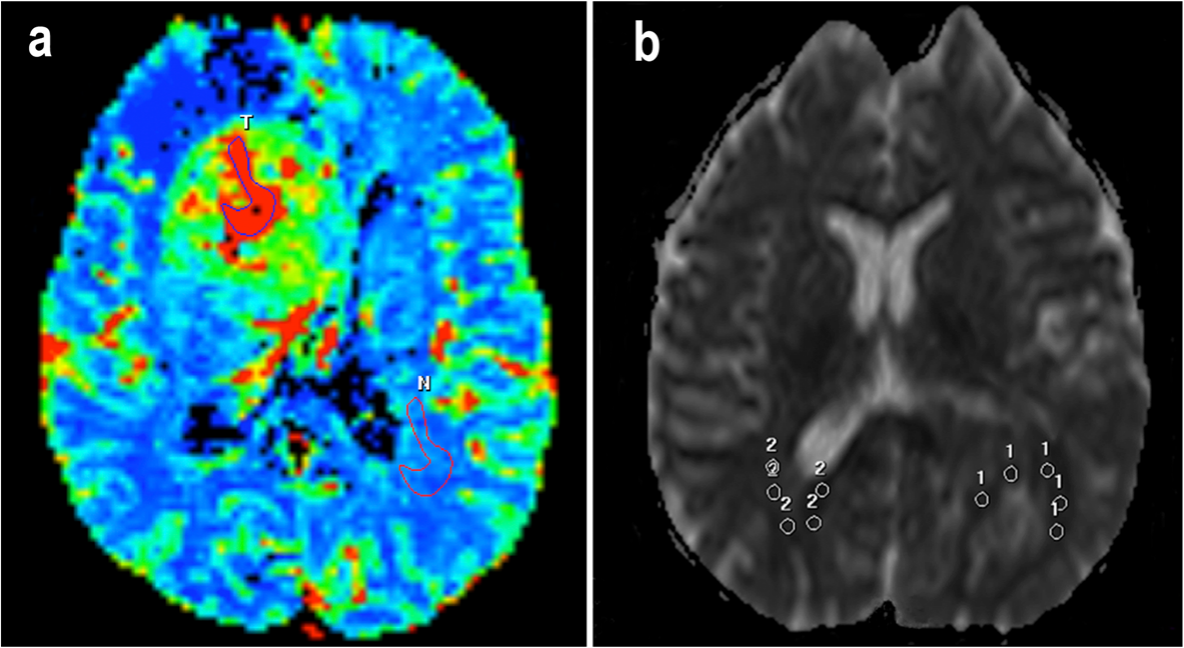
**Example of PWI and DWI assessment in cases of glioblastomas (grade IV). a**, Calculation of the rCBV ratio. The figure shows the ROI location covering the maximal values of CBV (T) in the parametric map. A similar ROI was placed in the contralateral white matter to normalize the image (N). This image corresponds to the conventional MRI images of Figure 1. **b**, Calculation of rADC. Example of five different ROIs placed within the solid area of the tumor in the ADC map (represented by number 1) and in the contralateral healthy area (represented by number 2). The MRI features of this case are shown in Figure 2.

The relative Cerebral Blood Volume (rCBV) was calculated using PWI (Figure 3) based on a region of interest (ROI) centered on the highest tumor rCBV value in the parametric map. This ROI was drawn as large as possible in an attempt to include all voxels with the highest and similar values of CBV. Unprocessed perfusion images and conventional post-gadolinium T1-weighted MRI images were used to ensure that ROIs were not placed over blood vessels. Tumor CBV was normalized to contralateral white matter CBV, on which an ROI of the same dimensions was drawn.

The relative Apparent Diffusion Coefficient (rADC) was calculated using DWI (Figure 3). Five different round-shaped ROIs ranging from 9.1 mm^2^ to 9.7 mm^2^ were placed in the solid tumor area. A further five ROIs with the same dimensions were placed in the contralateral normal cerebral area. The rADC was defined as the ratio of averaged ADCs between tumors and normal areas [18].

The Cho/Cr, NAA/Cr, Cho/H_2_O, and NAA/H_2_O ratios and the presence or absence of Lip or Lact were measured by MRS at long and short TE. We included the water peak as an internal reference following previously published data that report this approach to be a robust method for standardization [19]. Metabolic peak positions were assigned as follows: Cho, 3.22 ppm; Cr, 3.02 ppm; NAA, 2.02 ppm; Lip, 0.5-1.5 ppm. Lact (1.33 ppm) was identified as an inverted doublet at 144 ppm.

All variables obtained were assessed by consensus of two expert neuroradiologists with more than 10 years of experience (JG and PF).

### Statistical analysis

In the univariate analysis, continuous variables were assessed using the Mann–Whitney test and qualitative variables using a two-tailed Fisher exact test.

A multivariate logistic regression model was applied to assess the combined and independent values of predictor variables. We used a forward stepwise selection procedure with p-to-enter and p-to-remove value thresholds of p < 0.05 and p > 0.01 and a cutoff value of 0.5. Sensitivity, specificity, positive predictive value, negative predictive value, and a Receiver Operating Characteristic (ROC) curve were obtained for the predictor variables.

Statistical procedures were performed with SPSS version 13.0 (SPSS Inc, Chicago, Illinois, USA).

## Results

### Univariate analysis

The rCBV ratio could not be obtained in five patients owing to magnetic susceptibility artifacts resulting from an extensive tumor hemorrhage or a poor adjustment to the gamma curve.

Values of rADC were not calculated in five patients because of the presence of extensive necrosis. In this situation, there was not enough solid area to place the ROIs without partial volume effects in the necrotic region. Quantitative MRS results were not taken into consideration in 20 cases, owing to the poor quality of the spectra. In 24 patients, at least one of the metabolic ratios was missed, because the internal references (Cho or H_2_O) or the metabolite peaks of Cho and/or NAA could not be measured.

The presence of the MRI features was significantly greater in high-grade tumors (p < 0.0001). Statistically significant differences were found in rCBV (p < 0.0001), rADC (p < 0.0001), and the NAA/Cr (p = 0.005) ratio at a short TE and in the Cho/Cr ratio (p = 0.008) at a long TE (Table 2). The Lip peak was significantly present in high-grade tumors (p < 0.0001) (Table 2). The variables enhancement and necrosis showed the highest Odds Ratio (OR) for classifying high-grade tumors (55.42 and 23.82, respectively) (Table 3).

**Table 2:** **Comparison of perfusion-weighted, diffusion-weighted, and magnetic resonance spectroscopy variables between low-grade and high-grade tumor groups**

	**Low-grade tumor**				**High-grade tumor**				
**Variable**	**n**	**Range**	**Mean**	**SD**	**n**	**Range**	**Mean**	**SD**	***P*** **Value**
rCBV	29	0.00-13.61	2.09	3.17	95	0.51-19.18	5.75	4.10	<0.0001
rADC	30	0.81-2.55	1.74	0.44	94	0.43-2.63	1.17	0.38	<0.0001
NAA/Cr^a^	23	0.00-11.73	2.39	3.00	64	0.00-42.26	7.28	9.12	0.005
Cho/Cr^b^	24	0.95-21.43	3.56	5.34	70	0.81-77.46	7.95	14.88	0.008
Lipids	26	N/A	N/A	N/A	83	N/A	N/A	N/A	<0.0001
NAA/Cr^b^	24	0.00-11.61	1.53	2.28	71	0.00-20.36	1.49	3.15	NS
Cho/Cr^a^	23	0.07-9.30	2.00	2,13	65	0.00-30.15	3.24	4.91	NS
Cho/H_2_O^a^	19	3.02×10^−4^-5.88×10^−3^	6.60×10^−4^	1.29×10^−3^	63	0.00-6.18×10^−3^	4.21×10^−3^	8.03×10^−4^	NS
NAA/H_2_O^a^	20	0.00-3.28×10^−3^	6.67×10^−4^	7.85×10^−4^	63	0.00-1.73×10^−2^	1.00×10^−3^	2.20×10^−3^	NS
Cho/H_2_O^b^	17	2.10x10^−4^-3.98×10^−3^	8.39×10^−4^	9.00×10^−3^	64	4.74×10^−4^-8.27×10^−3^	8.34×10^−4^	1.23×10^−3^	NS
NAA/H_2_O^b^	18	0.00-1.01×10^−3^	3.12×10^−4^	2.74×10^−3^	67	0.00-6.86×10^−3^	3.98×10^−4^	1,18×10^−3^	NS
Lactate	26	N/A	N/A	N/A	83	N/A	N/A	N/A	NS

Cho, choline; Cr, creatine; NAA, N-acetyl-aspartate; N/A, not available (qualitative variables); NS, not significant; rADC, relative apparent diffusion coefficient; rCBV, relative cerebral blood volume.

^a^TE = 23 ms.

^b^TE = 144 ms.

**Table 3:** **Odds ratios obtained from the univariate analysis of the magnetic resonance variables with significant differences between low-grade and high-grade tumor groups**

**Variable**	**OR**	**95% CI**	***P*** **value**
Enhancement	55.42	15.58-197.15	<0.0001
Necrosis	23.82	8.43-67.35	<0.0001
Neovascularization	7.80	2.53-24.02	<0.0001
Edema	7.43	3.02-18.26	<0.0001
Hemorrhage	13.85	3.93-48.77	<0.0001
rCBV	1.68	1.23-2.19	<0.0001
rADC	0.05	0.01-0.16	<0.0001
NAA/Cr^a^	1.21	1.03-1.42	0.02
Cho/Cr^b^	1.05	0.97-1.14	0.22
Lipids	9.80	3.56-26.96	<0.0001

CI, confidence interval; OR, odds ratio; Cho, choline; Cr, creatine; NAA, N-acetyl-aspartate; rADC, relative apparent diffusion coefficient; rCBV, relative cerebral blood volume.

^a^TE = 23 ms.

^b^TE = 144 ms.

### Combination of the variables obtained by conventional MRI

**Figure 4:**
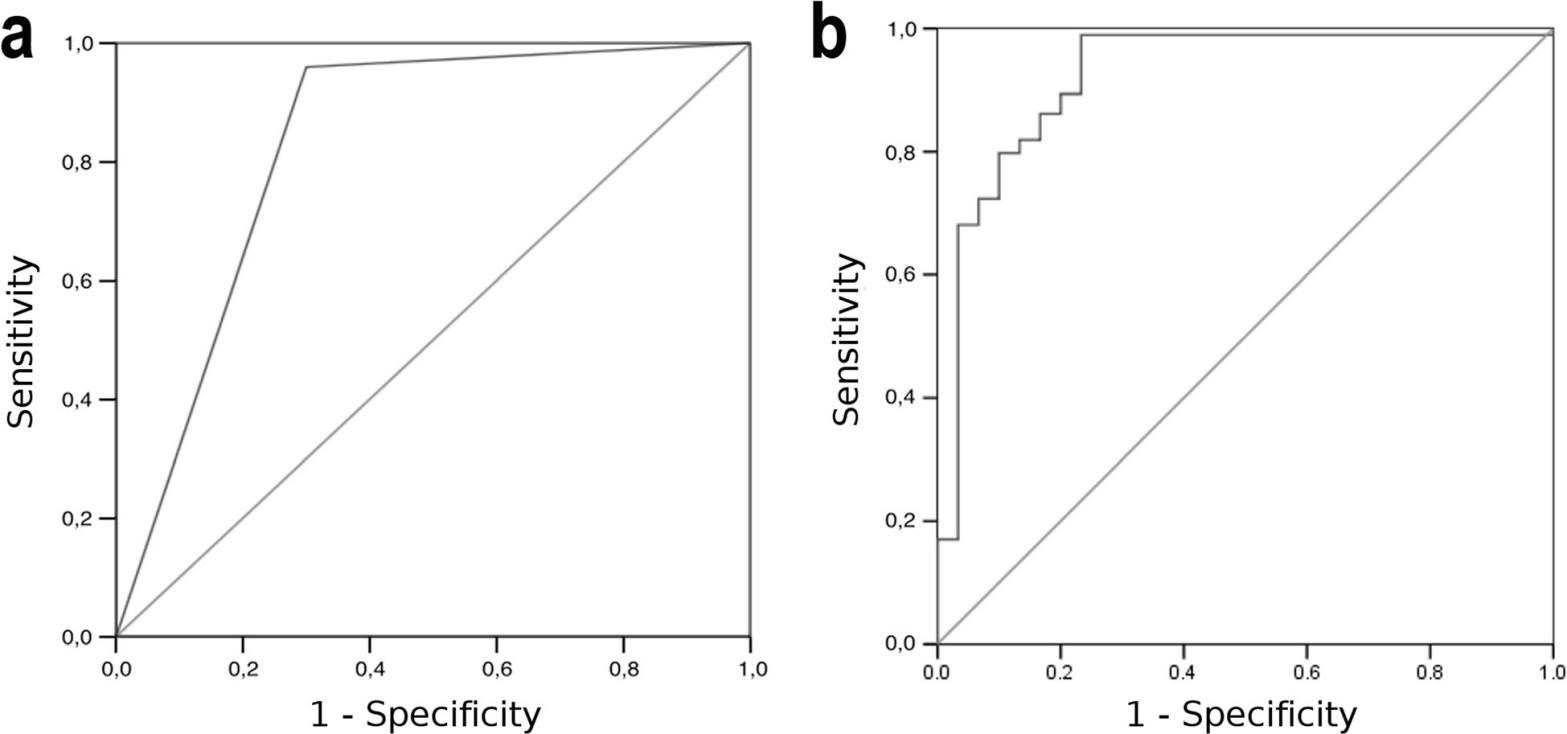
**Receiving operating characteristic curves of the combination of MR imaging features alone (a) or associated with PWI and DWI parameters (b) for grading of primary brain tumors.**

The imaging features identified as independent predictors of tumor grade were enhancement (OR, 23.37; 95% Confidence Interval -CI, 5.85-93.25) and necrosis (OR, 9.04; 95% CI, 2.61 -31.25). The sensitivity and specificity for the identification of high-grade tumors with these two features combined were 95.9% and 70%, respectively (Table 4). The Area Under the receiver operating characteristic Curve (AUC) was 0.890 (Figure 4).

**Table 4:** **Variables with independent predictive value obtained from combining different magnetic resonance techniques (multivariate logistic regression) in primary brain tumors**

**MR Technique**	**N**	**Predictor variables**	**OR**	**95% CI**	***P***	**Coefficient (β)**	**Standard error**	**AUC**	**Sensitivity (%)**	**Specificity (%)**	**PPV (%)**	**NPV (%)**
MRI	129	Enhancement	23.37	5.85-93.25	<0.0001	3.15	0.71	0.890	95.9	70	91.3	84
		Necrosis	9.04	2.61-31.25	0.001	2,20	0.63					
MRI	71	Enhancement	11.63	2.35-58.82	0.003	2.46	0.81	0.871	98.2	46.7	87.3	87.5
PWI												
DWI												
		Necrosis	8.48	1.95-37.04	0.005	2.13	0.77					
MRS												
MRI		Enhancement	16.95	3.89-71.43	<0.0001	2.83	0.75					
PWI	120	Necrosis	5.40	1.32-21.74	0.019	1.69	0.72	0.923	98.9	75.9	92.8	95.6
DWI		rADC	0.22	0.50-0.97	0.045	−1.51	0.75					

AUC, area under the receiver operating characteristic curve; CI, confidence interval; DWI, diffusion-weighted imaging; MRI, magnetic resonance imaging; MRS, MR spectroscopy; N, number of cases from which each classifier was constructed; PWI, perfusion-weighted imaging; rADC, relative apparent diffusion coefficient; OR, odds ratio; PPV, positive predictive value; NPV, negative predictive value.

### Combination of the variables obtained by conventional MRI, PWI, DWI, and MRS

This multivariate logistic regression analysis identified only enhancement (OR, 11.63; 95% CI, 2.35-58.82) and necrosis (OR, 8.48; 95% CI, 1.95-37.04) as independent predictors (Table 4). The sensitivity and specificity of these variables in grading brain tumors was 98.2% and 46.7%, respectively, and the AUC was 0.871. Thus, advanced variables seemed not to provide additional predictive value. However, it is noteworthy that one or more variables were missing in 58 cases, thus considerably reducing the sample size and the power of the analysis.

### Combination of the variables obtained by conventional MRI, PWI, and DWI

As most missing data corresponded to MRS, we conducted another analysis excluding the MRS–related variables. In this case, the number of cases remaining was 120, and the variables identified as independent predictors of high-grade tumors were enhancement (OR, 16.95; 95% CI, 3.89-71.43), necrosis (OR, 5.40; 95% CI, 1.32-21.74), and low rADC values (OR,0.22; 95% CI, 0.50-0.97) (Table 4). The sensitivity and specificity obtained with these three variables were 98.9% and 75.9%, respectively. The AUC was 0.923 (Figure 4).

### Multivariate analysis of gliomas

As most tumors in our series (91.5%) were gliomas (astrocytoma and oligodendroglioma), the multivariate logistic regression analysis was repeated in this histologic subtype. Independently of the variables included in the analysis, the only predictors of high grade were necrosis and enhancement, with a sensitivity of 97.6% and specificity of 76% when the conventional MRI, PWI, and DWI variables were combined (Table 5).

**Table 5:** **Variables with independent predictive value obtained from combining different magnetic resonance techniques (multivariate logistic regression) in gliomas**

**Technique**	**N**	**Predictor variables**	**OR**	**95% CI**	***P***	**Coefficient (β)**	**Standard error**	**AUC**	**Sensitivity (%)**	**Specificity (%)**	**PPV (%)**	**NPV (%)**
MRI	118	Enhancement	58.82	9.35-333.33	<0.0001	4.06	0.93	0.940	97.8	76.9	93.7	90.9
		Necrosis	13.89	2.91-66.67	0.001	2,63	0.80					
MRI	67	Enhancement	25	3.60-166.67	0.001	2.46	0.99	0.940	96.2	64.3	91.1	81.8
PWI												
DWI		Necrosis	8.26	1.53-43.48	0.014	2.11	0.86					
MRS												
MRI	110	Enhancement	50	8.13-333.33	<0.0001	3.94	0.94	0.940	97.6	76	93.3	90.5
PWI												
DWI												
		Necrosis	13.89	2.91- 66.67	0.001	2.64	0.80					

AUC, area under the receiver operating characteristic curve; CI, confidence interval; DWI, diffusion-weighted imaging; MRI, magnetic resonance imaging; MRS, MR spectroscopy; N, number of cases from which each classifier was constructed; PWI, perfusion-weighted imaging; rADC, relative apparent diffusion coefficient; OR, odds ratio; PPV, positive predictive value; NPV, negative predictive value.

## Discussion

Conventional MRI constitutes the most used MRI technique in the assessment of primary brain tumors. However, higher accuracy is necessary when grading brain tumors [3,8]. Advanced MR techniques provide additional information related to histological features of the tumor, as grade of neovascularization, cellularity, and mitotic index [10,20,21]. The validity of conventional and advanced MR in grading tumors has been widely reported in the medical literature [22–26]. The significant differences we found for the variables using all the MR techniques analyzed (conventional MRI variables, rCBV, rADC, NAA/Cr at short TE, Cho/Cr at long TE, and presence of lipids) were consistent with those reported in the literature.

Few attempts have been made to combine different MRI techniques in grading tumors [3,4,8,27] and, to our knowledge, only the study by *Yoon et al*. [16] has combined conventional MRI with PWI, DWI, and MRS in a group of patients diagnosed with cerebral gliomas. That study showed that there were no significant differences in the diagnostic performances of any of those MR imaging techniques. Recently, *Caulo et al.* [28] has also analyzed the information provided by these advanced MR techniques in the assessment of tumor grade in gliomas but, in their analysis, ADC maps were only used to define different tumoral regions in order to guide ROIs’ placement. Thus, the ADC values were not calculated to differentiate grade of aggressiveness. In our prospective study, we analyzed a series of variables for conventional MRI and advanced techniques (PWI, DWI, and MRS) to determine whether a combination of techniques was better than conventional MRI alone and, unlike in *Caulo et al.* [28], we used ADC values to assess tumor grade. We found that two conventional MRI variables, enhancement and necrosis, were the only predictors of grade in primary brain tumors.

The fact that data were missing from our study (at least one variable in 58 cases) could affect the statistical power. However, excluding these cases—and including only patients with all the variables—could lead to a selection bias. The radiologist should analyze brain tumors based on histological features (extensive necrosis or hemorrhage) that impair evaluation with advanced MRI techniques. For example, when necrosis was extensive, the area of the solid part was much too small to calculate advanced MR parameters [29,30]. Most published articles obviate this situation by avoiding cases with at least one missing data obtained by different MRI techniques [3,4,8]. As missing data correspond to MRS in most cases (56 cases), we performed an analysis combining all the variables except MRS data and found that at least one item of data was missed in only 9 patients. As a result, enhancement, necrosis, and low rADC were predictors of high tumor grade, and these variables provided higher accuracy (sensitivity 98.9%; specificity 75.9%) than those obtained with the other combinations analyzed (only MRI variables or MRI, PWI, DWI, and MRS variables). However, the improvement was no more than modest compared with the results obtained by combining only MRI variables (sensitivity 95.9%, specificity 70%).

Previous studies that analyzed differences between tumor grades were limited to gliomas [3,4,8,31–33]. However, we included all the primary brain tumors in order to mimic conditions of clinical practice, in which the radiologist has to provide a presumptive diagnosis of malignancy before surgery, regardless of the histological type. In our series 8.5% of tumors were non-gliomas due to the lower frequency of these type of tumors. Nevertheless, we repeated the multivariate analysis including only gliomas, since these were the most frequent histological subtype in our series. Unlike other authors, we were unable to demonstrate any additional value of advanced MR over conventional MRI [3,8,34], possibly because of our approach in assessing MRI. We showed high sensitivity and specificity (97.8% and 76.9%, respectively) for necrosis and enhancement as they were the best predictor variables of MRI in grading gliomas based on the results of the multivariate analysis combining conventional MRI variables. Using conventional MRI criteria, other authors obtained lower values (sensitivity of 42.1%-93.3% and a specificity of 60%-75.0%), possibly as a result of different selection criteria for high-grade MRI criteria [3,4,8,31,32]. For example, signal heterogeneity of the lesions could be related to other variables, such as presence of hemorrhage or necrosis. Mass effect is inherent to any tumor and is not necessarily associated with the histological grade. Furthermore, the existence of ill-defined borders is not useful in certain cases, such as glioblastomas, which could show well-defined borders on MRI, and low-grade gliomas, which tend to have an infiltrating appearance [35]. Some authors globally assessed the MRI features without specifying the diagnostic value of each of these imaging variables [3,4,22]. In addition, it is important to note that studies with negative results are less likely to be published, despite being well designed and conducted [36] thus leading to publication bias and overestimation of the value of advanced MR techniques in previous studies.

Our study has several limitations. We performed single-voxel MRS instead multivoxel MRS, which more accurately assesses tumor heterogeneity [37]. However, single-voxel studies have certain advantages, such as low time requirements, quicker post-processing, and better field homogeneity in the volume of interest [25]. The fact that most missing data were spectroscopic variables could lead us to underestimate the added value of MRS over conventional MRI in grading brain tumors. The rADC and rCBV ratios were calculated by selecting ROIs. As in the case of single-voxel MRS, this approach may be prone to sampling error, thus reinforcing the importance of careful placement of the ROIs. To reduce the T1 leakage effects, we did not administer a preload of contrast agent. Although this method seems to be the most robust for the evaluation of brain tumors, a statistical validation has not been provided [38]. In addition, to minimize the T1 and/or T2 leakage effects [38], we applied a gamma-variate function as a correction algorithm and the analysis of the MR signal-intensity curves did not show in any case of our series a rising of postbolus signal above the prebolus baseline that indicates T1-leakage effect.

In our study, we have excluded patients with massive hemorrhage. This criterion may be interpreted as contradictory because we analyzed the presence of tumoral hemorrhage by conventional MRI. However, we decided not consider only that cases that could be interpreted as brain hematomas secondary to brain tumors since the assessment of all advanced MRI techniques in these patients was not possible due to magnetic susceptibility artifacts secondary to the big quantity of blood products.

The restricted size of our sample prevented us from dividing it into training and validation sets, which would constitute a more suitable design for our analysis. Consequently, the accuracy we report in grading brain tumors could be somewhat optimistic. Nevertheless, the conclusions obtained by combining different MRI techniques should remain unaffected. Our approach is widely reported, thus making our results comparable [3,4,8,11,39].

## Conclusions

Preoperative diagnosis of tumor grade by MRI could assist in treatment planning, which is essential in cases were a histological diagnosis cannot be made. Our work focuses on different types of primary brain tumors, since, in clinical practice, tumor grade is analyzed using MRI with no previous knowledge of histological type. An appropriate analysis of conventional MRI features enables primary brain tumors to be graded with high accuracy. The best results for the prediction of high-grade tumors were obtained by combining the variables enhancement, necrosis, and rADC. Only a slight improvement was obtained with respect to conventional MRI criteria combined with the only advanced MRI variable considered as predictive (rADC). No advanced MR variables seem to add value to conventional MRI alone in the determination of grade in gliomas.
